# Exploring Music-Based Interventions for Executive Functioning and Emotional Well-Being in Stroke Rehabilitation: A Scoping Review

**DOI:** 10.3390/neurosci5040041

**Published:** 2024-11-27

**Authors:** Camila F. Pfeiffer, Wendy L. Magee, Rebecca Fülöp, Travis C. Nace, Candela Castro, Agustina Iturri, Jimena Franceschi, Gabriela Echauri, Liliana Gassull, María Julieta Russo

**Affiliations:** 1 Music Therapy Department, ArtEZ Academy of Music, ArtEZ University of the Arts, PN7511 Enschede, The Netherlands; 2 Facultad de Humanidades, Ciencias Sociales y Empresariales, Universidad Maimónides, Buenos Aires C1405, Argentina; 3 Music Therapy, Boyer College of Music and Dance, Temple University, Philadelphia, PA 19122, USA; wmagee@temple.edu (W.L.M.); rebecca.fulop@temple.edu (R.F.); travis.nace@temple.edu (T.C.N.); 4 Music and Health Science Research Collaboratory, Faculty of Music, University of Toronto, Toronto, ON M5S 1K6, Canada; castrocande@gmail.com; 5 Hospital Universitario Austral, Pilar B1629, Buenos Aires, Argentina; agustina.iturri@gmail.com; 6 Servicio Neurología Cognitiva, Neuropsicología y Neuropsiquiatría, Centro de Rehabilitación, CR, Departamento de Rehabilitación, Fleni, Buenos Aires C1428AQK, Argentina; jfranceschi@fleni.org.ar; 7 Servicio de Rehabilitación y Cuidados Continuos, Centro Hirsch, Buenos Aires B1663FDC, Argentina; gabriela.echauri@gmail.com; 8 Independent Researcher, Buenos Aires C1428, Argentina; liligassull@gmail.com; 9 Instituto de Neurociencias (INEU) Fleni Consejo Nacional de Investigaciones en Científicas y Técnicas (CONICET), Buenos Aires C1060AAF, Argentina; julieta.russo@csantacatalina.com.ar

**Keywords:** stroke, music therapy, music-based interventions, executive functioning, emotional well-being, cognitive rehabilitation

## Abstract

Purpose: Stroke is one of the leading causes of disability with life-long implications requiring assessment and treatment of several functional domains. This review identifies the results from research into music-based interventions (MBIs), including music therapy (MT), for executive functions (EFs) and emotional well-being (EWB) in adults with stroke and highlights opportunities for clinical practice and future research. Methods: APA PsycInfo (EBSCOhost), and CINAHL (EBSCOhost) were searched, in addition to grey literature. Results: A total of 49 studies were included and encompassed experimental, analytic, and descriptive observational studies, and case reports, involving a total of 1663 participants. In total, 32 studies included MT interventions, and 17 were MBIs. EFs were an outcome in 20.41%, and EWB in 61.22% of studies, for which active interventions were the most utilized. Overall, 73.47% of the studies reported positive results. Conclusions: This scoping review indicates that music interventions can be beneficial for the improvement of different aspects of EFs and EWB at different stages of stroke recovery. Further research may benefit clinical practice by including standardized protocols, outcome and self-reported measures, and brain imaging data to determine the effects of interventions and support evidence-based decisions for treatment policies for stroke survivors.

## 1. Introduction

Stroke is a major health concern with a high incidence worldwide, affecting millions of people annually [1]. Stroke is clinically defined as a vascular injury of the central nervous system that can be caused by a wide range of risk factors and disease processes. It can impact any brain region to different extents and its sequelae will depend, among other factors, on the size and location of the vascular lesion [2]. As one of the leading causes of death and disability around the world, it commonly causes cognitive, motor, sensory, and mood dysfunctions that can be either transient or permanent [2,3]. Current evidence suggests that cognitive impairments are prevalent after stroke and often remain present over time [4,5]. Specifically, executive functions (EFs) play an important role in functional recovery as they encompass a set of interrelated cognitive processes of learning and applying knowledge to behavior, which involve attention control, planning, working memory, cognitive flexibility, problem-solving, decision-making, and goal-oriented behavior. These processes often work interdependently with one another to accomplish goal-driven tasks, concentrate, or solve unexpected challenges [6,7,8]. Behaviors characterized as “dysexecutive” are common to stroke and can entail diminished mental flexibility, speed of processing, attention control, and a lack of inhibition control that can potentially lead to risky decisions in harmful situations [4,9]. Early treatment is necessary to prevent these behaviors from becoming chronic [3,10]. Together with post-stroke depression, executive functioning is a strong predictor of a person’s functional status after rehabilitation [11] and can seriously compromise their return to independent work and social life. As executive and emotional disorders frequently co-occur in stroke survivors [12,13], there are some indications that cognitive functioning is influenced by the person’s emotional state [14].

Emotional well-being (EWB) encompasses a variety of components and is broader than the relative absence of negative emotional states such as depressive or anxious feelings. EWB entails the perception of positive functioning, life satisfaction, positive social relationships, a feeling of life balance, and a sense of purpose [15]. According to the working definition developed by the National Institute of Health (NIH), EWB is a multidimensional construct that describes “how positive an individual feels generally and about life overall. It includes both experiential features (emotional quality …) and reflective features (judgments about life satisfaction, sense of meaning, and ability to pursue goals …). These features occur in the context of culture, life circumstances, resources, and life course” [16], p. 16. EWB is also linked to psychopathology and health outcomes with a consensus that they are on a continuum; a positive perception of EWB has been shown to reduce the risk of death by nearly 20% [17]. Stroke survivors commonly face some type of emotional and mood disorders (e.g., fatigue, depression, lack of initiative, emotional incontinence, anxiety, feelings of loneliness, apathy) and experience a diminished quality of life [18,19,20,21,22,23]. This implies that after a stroke, people may have limited opportunities for experiencing EWB. Consequently, rehabilitation treatment continues seeking effective and meaningful interventions that contribute to functional and emotional recovery.

Music has long been applied in different forms to treat stroke sequelae, for instance, through music listening, group singing, exercising with pre-recorded music, or longer music therapeutic processes [24,25,26]. Overall, music holds a high potential for promoting health [27,28]. MT utilizes evidence-based interventions that aim to accomplish personalized goals and are carried out by credentialed music therapy professionals [29]. MT is usually a process that includes assessment, treatment, and evaluation of the client’s progress over time [30]. Other MBIs are protocols that study the therapeutic effects of music, which can be delivered by other caregivers, do not take place in a therapeutic relationship typical for MT, and may be prescribed or delivered in a single contact without evaluation or follow-up [30,31]. Accordingly, this review utilized the term music therapy for studies in which music therapists were involved in the delivery of the intervention, and music-based interventions for those in which no music therapists were involved. The latest Cochrane review on MBIs for persons with acquired brain injuries found that music interventions are beneficial to motor recovery, communication, and quality of life in stroke survivors. However, no strong evidence could be found on the benefits of music on cognitive and emotional outcomes and further research was recommended [27]. A growing body of studies investigated the effects of music interventions on cognitive and emotional rehabilitation after stroke and reported positive results specifically on mood, depressive syndromes, and quality of life [26,32].

Despite the rapid advances in the field, there remain, however, some limitations in the literature that this scoping review seeks to address. Our objective is to synthesize comprehensive knowledge of the current literature available on MBIs, including MT, in stroke rehabilitation targeting EF and EWB. The review seeks to identify the types of interventions used to address these domains, the outcome measures utilized, and how gained data can be translated into opportunities that will guide clinical practice and future research. Given the specificity of the topic, a limited number of sources was expected; therefore, a broader scope on the two main outcomes was taken by considering factors that influence EWB and EFs, such as mood disorders, quality of life, and cognitive functions interdependent with EFs, such as attention and memory. A scoping review was considered the most accurate approach to obtaining a comprehensive understanding of the applications of music-based and music therapy interventions in stroke rehabilitation [33,34].

## 2. Materials and Methods

### 2.1. Search Strategy

The research question for this review was the following: What evidence exists about the benefits of music interventions to enhance executive functions and EWB in patients with stroke? The protocol was developed according to the methodological framework proposed by Arksey and O’Malley [33] and summarized by Colquhoun et al. [35], whereby the last optional step of stakeholder consultation was not conducted due to time limitations. We conducted and reported our search following the Preferred Reporting Items for Systematic Reviews and Meta-Analyses Extension for Scoping Reviews (PRISMA-ScR) [36]. The study protocol was registered at the Open Science Framework (https://osf.io/7u2f9, accessed on 14 November 2024). Health scientists and music librarians (TN, RF) developed detailed search strategies for the three databases included in this search using a combination of keywords and subject headings. The databases included in this search were PubMed (NLM), APA PsycInfo (EBSCOhost), and CINAHL (EBSCOhost). The search for PubMed (NLM) was translated for every database searched (see Literature search strategy in Supplementary Materials). The grey literature search included a clinical trials registry (clinicaltrials.gov), ProQuest Dissertations & Theses (PQDT) Global, and the TRIP Pro medical database (tripdatabase.com). The PubMed (NLM) search strategy was reviewed by the research team to check for accuracy and term relevancy, and all final searches were peer-reviewed by another librarian following the PRESS checklist [37]. The search spanned from the inception of each database to 25 October 2023, and was registered at the scholarly deposit of Temple University (https://scholarshare.temple.edu/handle/20.500.12613/8192, accessed on 14 November 2024). Duplicate studies were identified and omitted using the EndNote 20 duplicate identification strategy. The results from all databases were imported into Rayaan software [38].

### 2.2. Inclusion and Exclusion Criteria

This review included studies with a primary focus on (a) adults diagnosed with stroke; (b) quantitative, qualitative, and mixed methods studies; (c) music-based interventions including music therapy; (d) studies investigating outcomes influencing EFs, such as cognitive functioning, attention, and memory, and/or studies investigating EWB; and (e) outcomes influencing EWB, such as depression, anxiety, or quality of life. No language restrictions or time limits were applied to this study. Articles with the following criteria were excluded: (a) participants with other diagnoses; (b) book chapters, reviews, conference posters, or abstract-only papers; (c) interventions that did not include music; and (d) outcomes other than the emotional or cognitive domain (Table 1).

### 2.3. Screening and Data Extraction

Studies were screened by title and abstract by five blinded and independent reviewers (CC, AI, LG, GE, JF) (see description of the PRISMA flow diagram in the Supplementary Materials). This process was repeated for full-text article screening and article selection by the main author. A draft data extraction form was developed, discussed with, and revised by the research supervisors (MJR, WM) until the final version was completed. The full-text review was conducted by the main author, and data extraction was completed for the following: study characteristics (authors, year of publication, country, purpose of the study, target population, and settings), participant characteristics (age, gender, education, time post-stroke), characteristics of the MBIs (type of intervention, modality, description, interventionist, comparator, form of intervention, number of sessions, duration, and intervention period), and study outcomes (main outcomes and other primary outcomes, main findings, and result reporting).

### 2.4. Quality Assessment

This review considered including study designs with varying risks of bias due to the few published studies that included our specific variables of analysis. Under this argument, our quality assessment was based on the main objective of our study rather than on the study design. Only research that addressed our primary goal and offered reliable and practical information was reviewed. The appropriateness of the inclusion criteria, description of music interventions, outcomes measurements, and key results were assessed by blind pairs of reviewers who worked independently and with sufficient reliability to determine the validity of eligible studies. To reduce bias, studies were evaluated for inclusion based on selection criteria that were piloted to ensure their reliability and that they matched the primary goal.

In order to minimize bias, studies were assessed for inclusion using selection criteria that matched the main objective and that were piloted to check that they could be reliably applied [39].

## 3. Results

**Figure 1:**
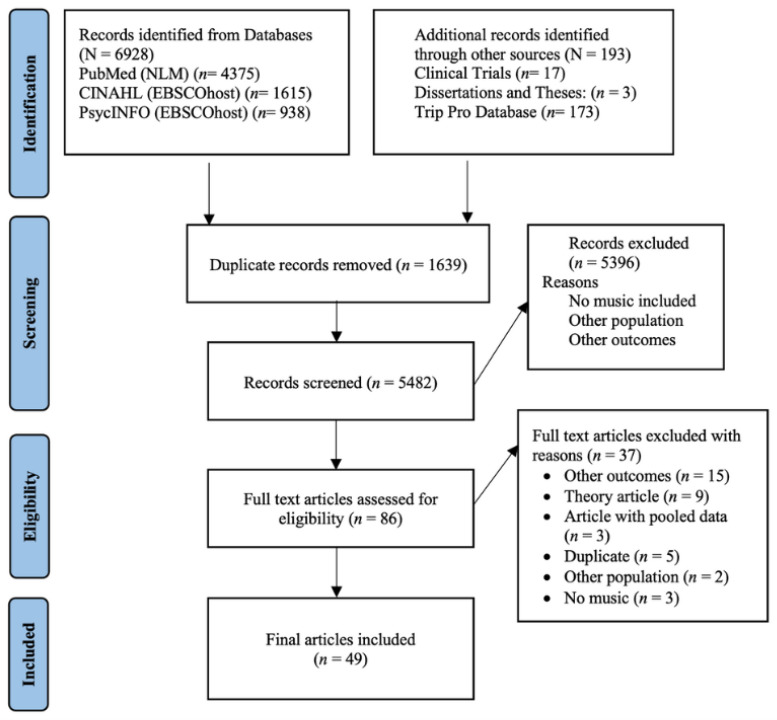
PRISMA flow diagram. The 49 papers included in this scoping review were published between 2000 and 2022, while the majority were published between 2016 and 2021 (61.22%, *n* = 30) (see Table 2). The first authors originated mainly from the United States (14.28%, *n* = 7), followed by the United Kingdom, Korea, and Italy (8.16%, *n* = 4 per country) (Table 3). Thirty-two were music therapy studies (65.3%) with the remainder being MBI studies (see Table 4). Study designs included 1 case report, 5 descriptive observational studies, 6 analytic observational studies, and 37 experimental/interventional studies, out of which 54% (*n* = 20) were randomized controlled trials (RCTs).

The search resulted in 7121 studies, and 1639 duplicate studies were found and omitted using the EndNote 20 duplicate identification strategy. This resulted in 5319 records, with an additional 163 records to screen from other methods, resulting in a total of 5482 records. A total of 5396 studies were excluded due to the lack of inclusion of music, other populations, or research outcomes. A full study analysis of the 86 remaining studies was conducted; ultimately, 49 were included in this review. Figure 1 provides the results of the search, which are described in the Supplementary Materials, and the study characteristics are given in Table 2 and the participants’ characteristics in Table 3.

### 3.1. Types of Music Interventions

As previously described, the included studies were categorized into two subgroups of MT and MBIs, depending on the participation of music therapists in providing the intervention. The majority of studies were MT studies (*n* = 32; 65.30%) (Table 4). Furthermore, two modalities of interventions were identified: (a) active interventions (music playing, singing, body movement to rhythm or music, songwriting, composing, improvising), and (b) receptive interventions (listening to music). In this regard, it was found that 75.51% (*n* = 37) of studies provided active interventions. Three studies defined the applied interventions as music therapy, although no music therapists were involved [46,57,58,82]. Considering that not all countries have music therapy training or credentialed professionals, the authors decided to still include these studies within the MT subgroup.

### 3.2. Active Music Interventions

A total of 71.43% (*n* = 35) of the studies applied active interventions. The majority were MT studies (*n* = 27), in which active music-making was utilized in various forms, for instance, singing and music-guided wheelchair exercises for well-being and motor recovery [56], playing musical instruments in a functional manner to rehabilitate upper limb movements [71], or functional music-making for recovery of body functions and quality of life [77]. Also, Neurologic Music Therapy (NMT) interventions [89] were used, such as “Rhythmic Auditory Stimulation” (RAS) to address gait rehabilitation, quality of life, and well-being [44,54]; “Music Attention Control Training (MACT)” and “Musical Executive Function Training (MEFT)” for attention, task-shifting skills, and executive control [55,60]; “Therapeutic instrumental music performance (TIMP)” for mental flexibility [52]; and “Melodic Intonation Therapy (MIT)” to address communication and EWB [87]. Furthermore, two studies investigated the participants’ experience of NMT treatment [80,83]. These studies involved an average of 34.34 participants (range 1–139), and an average of 13.16 music therapy sessions (range 1–30) with a duration from 20 to 60 min per session. Other active MT interventions utilized musical interactions in non-verbal settings [73]: instrumental playing and singing of pre-composed songs, improvising, musicalizing emotions, composing, relaxation exercises, music listening, and verbally sharing the musical experiences [57,61,62,63,64,66,67,68,78,86]. These interventions aimed to improve mainly mood and other components of EWB. Two studies utilized a therapeutic songwriting protocol targeting well-being, emotional distress, and self-concept, in which participants were assisted by the music therapist in identifying their salient thoughts and feelings and translating them into song lyrics [40,76]. Two other studies involved weekly participation in a community choir, in which simple vocal exercises and singing songs were used to improve socialization, communication skills, and EWB [47,82]. These studies involved an average of 25.63 participants (range 1–47), and an average of 9.75 sessions provided (range 1–30) with a duration ranging from 25 to 120 min per session.

Active MBIs (*n* = 8) primarily addressed functional motor recovery guided by rhythm, for instance, through the functional use of selected acoustic or digital musical instruments. In these interventions, the performance of hand, wrist, shoulder, arm, or body movements was matched to musical parameters, and hence, the physical movement required for the correct execution of playing the musical instrument trained the targeted movement by providing auditory feedback in the form of rhythmic patterns, harmonic progressions, or melodies [43,50,51,65,70,74,75]. Another study applied the performance of musical instruments, melodies, or songs in pairs, in synchrony, or in turns [84]. These studies involved an average of 45.73 participants (range 15–123), and an average of 33.27 sessions provided (range 10–96) with a duration ranging from 30 to 60 min per session.

### 3.3. Receptive Music Interventions

Receptive interventions were described in 28.17% (*n =* 14) of the included studies, with an equal number of MT and MBIs. In two of the MT trials, participants chose music of their preference to address cognitive recovery and mood [48,79], while in the others, the music was selected by the music therapist for a specific purpose, such as classical music to reduce anxiety [45] and Guided Imagery and Music (GIM) to improve quality of life [69], as well as instrumental music [81] and traditional Chinese music with acupuncture needling to reduce depression [59]. MBI studies investigated the impact of self-selected music listening on stroke rehabilitation [53] and on reducing anxiety [58], or mindful music listening to improve cognition and mood [41,42], while music selected by researchers was used to accompany a motor rehabilitation program [49,88], to improve disability and quality of life [85], or to compare the effect of different types of music on depression, sleep quality, mental state, and anxiety [46]. These studies involved an average of 55.43 participants (range 25–92), and provided an average of 36.79 sessions (range 1–96) with a duration ranging from 20 to 60 min per session.

### 3.4. Research Design and Other Study Characteristics

Table 4 includes data on research design, intervention comparators, interventionists, treatment settings, and units of delivery.

### 3.5. Study Outcomes

Executive function measures were included in 20.41% of the studies (*n* = 10), of which seven were MBI studies. Five of these studies used active interventions [50,51,52,65,75], and two used receptive MBIs [42,79]. Only two were MT studies [55,60], and lastly, one was a music-based assessment validation study [67]. Changes in cognitive flexibility, working memory, attention control, response inhibition, and information processing speed were mainly measured by three standardized outcome measures. The Trail Making Test (TMT) was the most utilized measure and was included in 70% (*n* = 7) of the studies, followed by the Digit Symbol Test (DST), included in 50% (*n* = 5), and lastly, the Stroop Test (ST) used in 30% (*n* = 3) of the studies. These measures were used in combination with each other or with other outcome measures detailed in Table 5. Only five of these studies investigated EFs as a primary outcome. EWB was the most investigated outcome across studies (*n* = 30, 61.22%), the majority of which were MT studies (*n* = 21). Active interventions were predominant to address this outcome (*n* = 26, 86.67%), and overall, 53.3% (*n* = 16) of studies investigated EWB as a primary outcome. Among the different aspects composing the construct of EWB, “mood” was the most widely investigated outcome, included in 66.67% (*n* = 20) of the studies, followed by “social interaction” included in 33.33% (*n* = 10). Other outcomes were overall well-being (four studies), affective state (three studies), self-concept, self-efficacy, emotional needs (two studies each), and perceived recovery, self-management, and coping mechanisms (one study each). Changes in EWB were documented by a great variety of outcome measures, counting 31 different tools. The most utilized outcome measures were the Profile of Mood States (POMS) (seven studies); semi-structured interviews and questionnaires and the Faces Scale (FS) (five studies); the Positive and Negative Affect Schedule (PANAS) and the Visual Analogue Mood Scales (VAMS) (four studies); and the Patient Health Questionnaire (PHQ-9) (three studies).

### 3.6. Outcomes and Measures for the Cognitive and Emotional Domains

Cognitive functioning was an outcome in 36.73% (*n* = 18) of studies and was the primary outcome in half of those. Improvements in attention, memory, and language were registered through nineteen different outcome measures, of which the most utilized were the Mini Mental State Examination (MMSE) (five studies), the Montreal Cognitive Assessment (MOCA) (two studies), and the Cognitive Linguistic Quick Test (CLQT) (two studies). Three studies included participants diagnosed with aphasia [69,79,87]. The majority of studies used MBIs (*n* = 10), of which six were active, and four were receptive. Nearly a third of all studies (*n* = 15) included outcomes within the emotional domain, in which depression (10 studies) and anxiety (7 studies) were the most prevalent aspects measured. The most utilized outcome measures to report changes in symptoms of depression were the Hamilton Depression Scale (HAMD) (three studies), the Beck Depression Inventory (BDI) (two studies), and the Hospital Anxiety and Depression Scale (HADS) (two studies). Improvements in anxiety were captured mainly through the Hospital Anxiety and Depression Scale (HADS) (three studies) and the State–Trait Anxiety Inventory (STAI) (three studies). The emotional domain was a primary outcome in 53.33% (*n* = 8) of those studies, among which anxiety and depression were equally distributed. MT and MBIs were used in equal frequency (*n* = 4 each), whereby almost all were of receptive nature (*n* = 7). All outcome measures used for the cognitive and emotional domains are detailed in Table 5.

### 3.7. Other Outcomes and Measures

Studies included other outcomes relevant to EFs and EWB, which are mainly those of quality of life (QoL) and the motor domain. QoL was included in 30.61% of studies (*n* = 15) and was a primary outcome in three studies. The most frequently utilized measures were the Stroke-Specific Quality of Life Scale (SSQOL) (five studies), the Health Survey Questionnaire (SF-36) (three studies), and the Stroke Impact Scale (SIS) (three studies). More than half of these studies (*n* = 8) applied music therapy interventions. Furthermore, the motor domain was included in 36.73% of studies (*n* = 18), and a primary outcome in 66.67% of those studies (*n* = 12). Recovery of hand and upper limb function was the most common clinical outcome (*n* = 12), for which improvements were documented by eighteen different outcome measures. The most utilized were the Fugl Meyer Scale (FMS) (four studies), the Action Research Arm Test (ARAT) (three studies), the Box and Block Test (BBT) (three studies), and the Nine-Hole Peg Test (9 HPT) (three studies). Only eight studies included music-based outcome measures, which mostly aimed to assess music cognition. The Montreal Battery of Evaluation of Amusia (MBEA) was included in two studies, and all other measures are specified in Table 5.

### 3.8. Results Reporting

The main outcomes were classified as positive, negative, or mixed based on a system proposed in another study [90]. Positive outcomes refer to studies in which the target outcome(s) improved for all participants. Negative outcomes referred to studies in which no treatment effect was observed following music intervention. Mixed outcomes referred to studies in which some participants made improvements and others did not. Positive results were reported in 73.47% of studies (*n* = 36), while 18.37% (*n* = 9) reported mixed results, 6.12% (*n* = 3) reported negative results, and one study did not report clinical results. Active music therapy interventions had a positive effect in 74.04% (*n* = 20). The long-term effect of the musical intervention was only reported in five of the nine studies that investigated this domain [40,42,43,50,51,71,79,82].

## 4. Discussion

This scoping review aimed to synthesize comprehensive knowledge of the available literature on MBIs, including MT, targeting the rehabilitation of executive functions and EWB of adult stroke survivors. Overall, the results indicated that stroke survivors improved in the investigated outcomes, as the predominance of positive or mixed results was high throughout the studies (94.95%). The wide variety of study designs and methodologies precluded critical appraisal and risk of bias. A data extraction sheet allowed the authors to understand each included study by identifying and presenting relevant information in different tables.

### 4.1. Summary of Main Results

The results indicated that MBIs improved various aspects of EFs, such as cognitive flexibility, attention control, divided attention, working memory, task shifting, working memory, and performance speed. The outcome measures most frequently utilized were the Trail Making Test (TMT) and the Digit Symbol Test (DST); however, mostly, more than one tool per study was utilized to assess EFs. The TMT comprises tests A and B, which, together, measure cognitive flexibility, visual attention, task shifting, and speed [91]. The DST measures verbal and short-term memory and can be used in two ways: the Forward Digit Span measures attention and immediate memory, while the Backwards Digit Span measures working memory and complex attention [92]. Together, these measures provide a standardized and relatively good estimate of EFs [93] that provides insights into the cognitive, functional, and emotional dimensions of functioning at all stages of stroke [94]. However, the psychometric properties of both measures have not yet been characterized in patients with stroke [7], nor have they yet been proven to significantly relate to everyday executive functioning, or noncognitive factors that can influence test performance and everyday performance [7,95]. Therefore, the ecological validity of these outcome measures requires further research.

EWB was a primary outcome in sixteen of the included studies, and music interventions applied were almost all active, including music playing, singing, and musical and rhythmic activities [43,57,61,62,68,78,86]; songwriting [40,76]; and singing in choirs [47,82]. Receptive interventions, such as mindful music listening [41,42] or listening to preferred music, were used to improve mood [26], and mood was the most investigated component of EWB [47,48,50,57,61,62,68,78,79,86]. Self-concept, perceived recovery, and social interaction were other components of relevance [40,48,76,86]. Improvements in EWB were identified by participants and their caregivers as one of the key benefits, specifically regarding mood, social interactions, and meeting emotional needs [41,47,48,63,70,80,83]. The Profile of Mood States (POMS) was the most frequently utilized standardized outcome measure, which is a widely used tool to assess mood states. The original scale measures six different dimensions of mood swings, including anxiety, anger, activity, fatigue, depression, and confusion. It has a fairly good internal consistency (0.63 to 0.96 Cronbach alpha rating), while the shortened version has similar psychometric properties [96]. There are several versions of the POMS; however, none have been validated in a stroke population. Despite the fact that music experiences are considered to be directly connected to cognitive functioning and emotional processes [26,97,98], only seven studies included music-based outcome measures. Similarly, although from a neurorehabilitation perspective, the mechanism for music’s effects is believed to be based on neuroplasticity, only three studies utilized neuroimaging techniques.

### 4.2. Findings on Music-Based Interventions

The synthesis of the results gained in this review indicates that MT and MBIs can positively influence different components of EFs and EWB. A wide variety of interventions were identified with the majority being active MT interventions, carried out mainly in groups and facilitated by certified music therapists. Interventions such as music-guided motor therapy, and the therapeutic performance of music, were consistently shown to improve cognitive functioning, specifically attention, memory, verbal memory, and EFs, 50–53, 76. Similarly, the goal-directed use of music in NMT interventions showed improvements in executive functioning [55,60]. The goal-oriented use of music may lead to positive results, as only 18.75% of goal-oriented active musical interventions yielded mixed or negative results. Other MT interventions, which included mainly music playing, musical improvisation, singing, and therapeutic songwriting, were mostly applied to address components of EWB [40,57,61,62,63,64,68,69,71,72,73,76,78,82,86]. Almost 40% of those studies yielded mixed results, which could suggest that interventions providing more room for creativity and that are more experience- and process-oriented might be suitable to address some components of EWB. There was a notable difference in the use of music when comparing active MT and MB interventions. In MBIs, recorded music or the metronome were auditory organizers of timed body movement or as motivators for motor activation, while digital musical instruments were used to train specific movements, mostly tracked by a computer. Although music was also used in a functional way in MT, almost no standardized music was utilized; instead, the music therapist created and facilitated the music in a personalized manner for the participant’s needs and therapeutic goals. The musical elements were carefully balanced and selected to achieve a therapeutic purpose. MT trials also used music in interactive and creative ways to address psychosocial needs through improvising, singing, playing musical instruments, and creating songs of personal meaning to the participants that reflected their inner thoughts and feelings. The music therapist provided the musical context, musical logic, structure, and strategic therapeutic use of music to drive the necessary change. Assessing the possible interrelation between EWB and cognitive functions was an aim of this scoping review. However, at this point, it is not possible to draw any conclusions from the selected studies about a possible correlation between the emotional state and the cognitive performance of stroke survivors.

The findings from active interventions are supported by research on music and the brain. Being actively engaged in music-making is defined by neuroscience research as one of the cognitively most demanding activities, due to its complex and multisensory characteristics, and its influence on cognitive, affective, and sensorimotor functions [97,98,99,100]. Making music has motivational salience in the activation of key limbic and reward-related brain structures [101] and involves a set of cortical and subcortical brain regions and networks that are not modulated by music [102]. Hence, music engages the brain at multiple levels, consequently altering the physiology and neurochemistry of the brain [103] and cortical pathways, positively affecting task performance across various domains [104]. Eventually, cognitive enhancements through active music-making might be explained by brain plasticity [104,105,106]. Lastly, from a therapeutic point of view, engaging in therapeutic and social relationships promotes self-esteem, enhances mood, and diminishes feelings of loneliness, isolation, and depression [63,80]. An active therapeutic space, in which social communication takes place within the shared activity of music, allows its participants to rehearse, experiment, and increase intra- and interpersonal skills [107,108]. Although receptive interventions may be cost-effective and easy to implement, only less than one-third of the studies examined the effect of listening to music (preferred and mindful music listening) for different purposes. While some studies yielded positive results for mood [79], anxiety [58], and depressive symptoms [69,88], other studies could not confirm these findings [42,46,53]. Despite the inconsistent results, participants and healthcare professionals identified receptive interventions as meaningful in meeting the emotional needs of participants [42,48]. Hence, evidence is still too inconsistent and insufficient to claim a positive effect or to recommend music listening to post-stroke emotional disturbances. Studies with a larger sample size and a clearer methodological protocol are needed to draw confident conclusions, for example, on the duration of the intervention.

Most studies utilized standard treatment or non-musical interventions as comparators, while only 14.28% used other musical or auditory interventions. Few studies described the intervention in detail, endangering possibilities for follow-up research and further investigation of the effect of specific music genres, styles, or elements on neurophysiological and neuropsychological outcomes. Moreover, despite available evidence on the emotional meaningfulness and involvement of the brain reward system with participants’ preferred music, few studies have specified the type of music utilized and if it was self-selected or experimental music. To elucidate the effect of specific musical elements or genres, investigations on MBIs should include musical comparators. Lastly, at this point, it is not possible to provide any recommendations on a specific dosage of MBIs. With a total median of 15.5 sessions provided across studies, the high number of positive results is encouraging, although a longer period of intervention was often recommended. An extended period of interventions may support brain plasticity [106] and might elicit more detailed and consistent changes in the behavior of stroke survivors. To verify the permanence of change and improvements, long-term effects should be measured whenever possible. Only a small number of researchers conducted longitudinal studies; the high frequency of withdrawal in clinical settings and funding for continuous research are often challenges in accomplishing the study of the long-term effects of treatment.

More than half of the interventionists were credentialed music therapists and almost one-third of studies did not specify who provided the interventions. Little information was found on the role and relevance of the interventionist, or the therapeutic relationship to treatment outcomes. This is noticeable since a therapeutic relationship partially defines music therapy as an intervention [29]. Currently, there is increased collaboration between music therapists and neuroscientists, who seek to contribute with evidence on the efficacy of the standardized use of music to drive behavioral changes. The establishment of music therapy as an evidence-based intervention and its positioning in interdisciplinary medical treatments and neuroscience research has been core to music therapy research in the past few decades [89,109,110]. This specific focus might explain why little is reported about the influence of the professional delivering the intervention. Nonetheless, research suggests that the involvement of music therapists is relevant to the efficacy of musical interventions [27,30]. The themes highlighted by some authors and the participants’ descriptions of their experiences in music therapy support these findings and highlight the value of music therapy for meeting social and emotional needs, facilitating emotional expression, and building new relationships [47,48,63,73,78,80,83,87]. Further investigation on the role and the influence of the therapeutic alliance, and the specific role of the music itself for therapeutic processes and outcomes might help to further differentiate the accurate application and efficacy of MT from other MBIs.

Lastly, further research of higher methodological rigor has been recommended to gather trustworthy evidence on the benefits of music [25,26,27,32,111,112]. Interestingly, from 2016 onwards, mixed methods have been used, which complement evidence gained from quantitative outcome measures with experiences from clients, caregivers, and multidisciplinary treatment teams [47,62,64,71,73,80]. Standardized outcome measures are not always the most suitable for stroke survivors with severe disability and may fail to capture behavioral changes in the limited timeframe of a research protocol. Moreover, perceived improvement, motivation, and interrelation aspects, commonly not considered in standardized measures, may play a significant role in recovery. Therefore, mixed methods studies may be an optimal way to gather multilayered evidence for a complex intervention for complex conditions.

### 4.3. Study Limitations

Although several of the database searches included grey literature, no additional hand search of unpublished grey literature was conducted. All sources are published studies in English, and searches in dissertation repositories might have contributed to unpublished work conducted in non-English-speaking countries and cultures. The authors preserved a broad perspective on the topic, which inevitably led to great heterogeneity in the study designs and interventions. EFs and EWB were investigated with different priorities (primary, secondary, together with motor or aphasia rehabilitation), across different stages of recovery, and in diverse settings. Thus, only a few primary outcomes have been studied repeatedly.

### 4.4. Recommendations for Future Directions

Music has been gradually and robustly proven as beneficial to stroke rehabilitation. Although evidence is growing, further research is needed in the domains of cognition and EWB. The inconsistencies in the descriptions of relevant information in the studies included in this review impede the translation of gained evidence into clinical practice guidelines and policies. Adopting existing standardized reporting guidelines is key to clear and consistent documentation of studies and their outcomes, which will allow for consistent communication across different disciplines and for drawing clear practical conclusions [113]. The following recommendations result from the authors’ conclusions, which will hopefully be useful to guide future research and clinical directions.

### 4.5. The Client Population and the Treatment Setting

Demographics, including the cultural background and the level of education of participants, and details on the diagnosis (e.g., time since stroke, localization, comorbidities, musical experience) are necessary to identify how music influences specific stroke sequelae. The stage of recovery needs to be described, as acute and subacute rehabilitation may include intensive interdisciplinary inpatient treatment [114], while persons in the chronic stage might receive either outpatient, home-based, or no treatment at all [115]. A clear picture of the participant’s functional baseline will help to better identify the meaningfulness of a new treatment protocol.

### 4.6. The Music Intervention and the Interventionists

A detailed description of the intervention, the utilized music (client-preferred, experimenter-selected, specific musical elements), and the dosage including a clinical rationale and scientific underpinning is recommended. Research on the effect of specific musical elements, genres, or the comparison of music interventions is lacking and may lead to a better understanding of the neuroplastic effects specific to music-based rehabilitation [49,88]. It is recommended to describe the role of the interventionist and to examine to what extent the therapeutic relationship could influence functional outcomes. This will allow a clearer differentiation between music therapy and other MBIs and guide institutional decisions on the interventions needed.

### 4.7. Research Design

Future studies should consider applying mixed methods designs and longitudinal studies. While high-quality quantitative designs, such as RCTs, will continue providing evidence to identify the most effective interventions, other valuable information can be gathered by documenting the participant’s experience that cannot be captured by standardized measures, such as the significance of the interventions for social and emotional needs. A bigger sample size, multicentered studies, and the inclusion of brain imaging techniques will help determine the specific effect of music interventions on the person with stroke and translate this evidence to treatment policies.

### 4.8. The Need to Build a Body of Knowledge

Conducting follow-up studies will help to measure the effect and efficacy of MBIs on specific outcomes. Standardized protocols and the consistent use of outcome measures, including music-based measures, brain imaging data, and self-reported measures, would help explain the different neurological and behavioral patterns, supporting the use of specific and targeted music interventions. Different studies suggested a direct relation between the intrinsic motivation caused by the engagement in music interventions, and the functional improvement [51]. Research is needed to identify the specific role of music in combined treatment, for instance, by investigating the intersections between functional outcomes, adherence to treatment, motivation, and music. A growing body of knowledge [116,117] will help to build a rationale for the clinical relevance and application of specific music interventions.

## 5. Conclusions

This review has found that MBIs, including music therapy, can be beneficial for treating executive functions and the EWB of adult stroke survivors at different stages of recovery. These improvements could probably be generalized to other domains considered important in the context of a rehabilitation plan (e.g., motor, cognition, language, and functionality). Although no direct relationship could be found at the intersections of executive functioning, EWB, and music, the literature suggests that the intrinsic motivation caused by engaging in different music interventions, as well as the goal-directed use of music, can lead to functional improvement. Future research would benefit from studying these intersections through high-quality research including standardized interventions and outcome measures, self-reported measures, and brain imaging data that will help determine the effect of specific interventions for specific outcomes. Additionally, exploring the role of a therapeutic relationship in the rehabilitation process will lead to a more detailed differentiation between music therapy and MBIs, and guide evidence-based decisions for treatment policies of stroke survivors.

## Figures and Tables

**Table 1:** Eligibility criteria.

	Inclusion	Exclusion
1. Participants	Adults with acute, subacute, and chronic stroke, with or without aphasia or other comorbidities. Healthy participants (caregivers, healthcare providers) included in interviews or questionnaires.	Studies including only participants with diagnosis of acquired brain injury other than stroke (e.g., traumatic brain injury, disorders of consciousness).
2. Study design	Experimental/interventional studies, analytic observational studies, descriptive observational studies, validation of music-based assessment studies, Ph.D. dissertations or theses.	Systematic or other types of literature reviews, pre-clinical studies, conference abstracts, or posters with no available full-text article.
3. Interventions	Music therapy interventions. Active: music-making (acoustic or digital musical instruments), singing, songwriting, musical improvisation, composing, verbally sharing experiences with the therapist. Receptive: music listening; multidisciplinary: combining music therapy with other health disciplines. Neurologic Music Therapy techniques. Music-based interventions include singing, choir participation, music-guided movement, sonification, music technology, and music listening. Provided in individual or group sessions. No limitations to interventionists.	Therapeutic interventions or standard treatment that did not include a music intervention.
4. Outcomes	Papers in which (one of) the primary and/or secondary outcome(s) was executive functions (task shifting, attention, memory, verbal memory, flexibility, information processing) and/or emotional well-being, as conceptualized by Park et al. (2023) [16], (such as positive affect, life satisfaction, quality of life, and sense of meaning), and influencing emotional components, such as depression and anxiety. If there was no clear distinction between primary and secondary outcomes, studies with outcomes in one or both domains were included.	Outcomes other than in the cognitive or emotional domain.

**Table 2:** Study characteristics.

Citation	Location	Study Design	Purpose/Objective	Target Population	Setting
[40]	Australia	Experimental/interventional study	To determine the size of the effects and feasibility of a therapeutic songwriting protocol for inpatients and community-dwelling people with ABI or SCI	Chronic SCI (24), ABI (23)	Rehabilitation Center and Community
[41]	UK	Analytic observational study	To investigate participants’ experiences of mindful music listening	IS (27 RH, 40 LH, 4 other)	Acute Stroke Unit
[42]	UK	Experimental/interventional study	To assess the feasibility and acceptability of a novel mindful music listening intervention	Acute and Early Subacute IS (27 RH, 40 LH, 4 other)	Acute Stroke Unit
[43]	Sweden	Experimental/interventional study	To assess whether multimodal interventions based on rhythm-and-music therapy or horse-riding therapy could increase the perceived recovery and functional improvement in the late phase after stroke	Chronic Stroke (58 RH, 65 LH)	Community
[44]	Korea	Experimental/interventional study	To investigate the effect of intensive gait training with RAS on postural control and gait performance in individuals with chronic hemiparetic stroke	Chronic Hemiparetic Stroke	Hospital
[45]	Turkey	Experimental/interventional study	To investigate the effect of one session of MT on anxiety	Stroke (31), Healthy (53)	Rehabilitation Center
[46]	Turkey	Experimental/interventional study	To investigate the effects of listening to different types of music on depression, sleep quality, mental state, and anxiety post-stroke	Early Subacute Stroke	Hospital
[47]	New Zealand	Descriptive observational study	To explore the experiences of and factors influencing participation in CST by people with stroke or PD and their significant others	Chronic Stroke (8), PD (6), Healthy (9)	Community
[48]	Finland	Descriptive observational study	To gain more insight into the therapeutic role of music listening	Acute stroke (20), Healthy (5)	Rehabilitation Center
[49]	Greece	Experimental/interventional study	To assess the effects of an exercise rehabilitation program with experiential music for clinical recovery	Early Subacute Stroke	Rehabilitation Center
[50]	Canada	Experimental/interventional study	To investigate the effects of MST in chronic stroke on motor, cognitive, and psychosocial functions compared to GRASP	Chronic Stroke	Community
[51]	Spain	Experimental/interventional study	To test the effectiveness of adding MST to a standard rehabilitation program in subacute stroke for motor, cognitive, and QoL domains	Early and Late Subacute Stroke (32 IS, 7 HS)	Rehabilitation Center
[52]	Canada	Experimental/interventional study	To investigate the effects of TIMP with and without motor imagery on upper extremities of individualswith chronic, post-stroke hemiparesis	Chronic Stroke	Community
[53]	Australia	Experimental/interventional study	To investigate the feasibility and impact of music listening in addition to standard care	Acute Stroke (33 IS, 5 HS)	Acute Stroke Unit
[54]	South Korea	Experimental/interventional study	To test the effect of a theory-driven music exercise intervention on stroke survivors’ physical functioning, psychosocial functioning, and QoL	Chronic Stroke (20 IS, 16 HS; 15 RH, 17 LH, 1 both)	Community
[55]	Canada	Experimental/interventional study	To investigate the potential effectiveness of music-based cognitive rehabilitation for adults with chronic ABI	ABI (5 Chronic Stroke, 1 Tumor, 9 TBI)	Hospital
[56]	Korea	Experimental/interventional study	To evaluate the effects of combined music–movement therapy on physical and psychological functioning	Acute/Early Subacute Stroke	Rehabilitation Center
[57]	Korea	Experimental/interventional study	To investigate the effects of MT on the mood of stroke patients	Subacute Stroke	Rehabilitation Center
[58]	USA	Experimental/interventional study	To determine if listening to music may reduce anxiety experienced by stroke patients during acute rehabilitation	Stroke (36 IS, 5 HS)	Rehabilitation Center
[59]	China	Experimental/interventional study	To evaluate the clinical efficacy and safety of five-phase MT in patients with depression after stroke	Subacute Stroke with Depression	Hospital
[60]	USA	Experimental/interventional study	To determine the feasibility of an MEFT intervention to address task-shifting skills in adults with ABI and to obtain preliminary evidence of intervention effect on task shifting	Chronic Stroke	Rehabilitation Center
[61]	United Kingdom	Experimental/interventional study	To examine the effect of MT on mood states in patients with acquired and complex neuro-disabilities	ABI (5 MS, 5 TBI, 4 stroke)	Residential Rehabilitation Facility
[62]	Canada	Analytic observational study	To evaluate change in mood and pain following a single MT session; to explore the impact of an MT program on mood, pain, and satisfaction from the perspective of the patient, family, and staff	Stroke (14), Healthy (26)	Acute Stroke Unit
[63]	USA	Experimental/interventional study	To evaluate whether MT is effective in enhancing a patient’s mood, social interaction, and involvement in therapy during acute rehabilitation	ABI	Rehabilitation Center
[64]	USA	Analytic observational study	To refine MULT-I and compare its biologic and behavioral effects with that of an HEP	Chronic Stroke with Hemiparesis	Hospital
[65]	Korea	Experimental/interventional study	To investigate the effects of CMDT combined with AMST utilizing rhythmic cues on cognitive function in patients with stroke	Chronic Stroke (11 RH, 19 LH)	Hospital
[66]	Argentina	Experimental/interventional study	To explore the clinical utility of the Screening of Music Cognition to basic cognitive skills of adult patients with right hemisphere stroke	Late Subacute Stroke (15), Healthy (30)	Neurorehabilitation Center
[67]	Argentina	Experimental/interventional study	To develop a music-based scale to assess the cognitive functions and mood of adults with ABI and determine its psychometric properties in terms of internal consistency, reliability, and concurrent validity	Late Subacute Stroke (10), TBI (10), Healthy (24)	Neurorehabilitation Center
[68]	Poland	Case report	To identify the impact of individual music therapy on mood, anxiety, emotional control, acceptance of illness, coping style, and other parameters of health psychology	Stroke	Neurorehabilitation Center
[69]	Poland	Experimental/interventional study	To determine whether MT during neurorehabilitation can positively influence QoL after a stroke	Late Subacute Stroke (36 RH, 23 LH, 2 BS; 49 IS, 12 HS)	Neurorehabilitation Center
[70]	Sweden	Descriptive observational study	To explore the experiences of stroke survivors who participated in a group-based multimodal rehabilitation program based on rhythm and music	Chronic stroke	Community
[71]	US-	Analytic observational study	To investigate the long-term post-stroke effect on upper limb recovery of the MULT-I	Chronic Stroke with Hemiparesis (5 IS, 5 HS)	Hospital
[72]	Italy	Experimental/interventional study	To evaluate the effects of active MT and SLT compared to SLT alone in stroke patients with chronic aphasia	Chronic Stroke	Rehabilitation Center—Outpatients
[73]	Italy	Experimental/interventional study	To examine if RAMT can improve psychological outcomes and communicative/relational aspects, as well as fine and gross motor skills, in particular in upper extremities	Early Subacute Stroke	Hospital–Inpatients
[74]	Italy	Experimental/interventional study	To evaluate the efficacy of a music-based sonification approach on upper limb motor functions, QoL, and perceived pain	Early Subacute Stroke (36 RH, 30 LH)	Rehabilitation Center—Inpatients
[75]	Spain	Experimental/interventional study	To assess the motor, cognitive, emotional, and neuroplastic effects of MST	Chronic Stroke (11 RH, 9 LH)	Hospital
[76]	Australia	Descriptive observational study	To examine changes in self-concept, distress, well-being, and functional skills through songwriting	Late Subacute Stroke (1 RH, 1 LH, 1 MT), ABI (3)	Rehabilitation Center—Inpatients
[77]	Sweden	Descriptive observational study	To estimate the effects of FMT on several body functions in patients with chronic stroke and PD	Chronic Stroke (5 RH, 5 LH), PD (10)	Outpatients Location
[78]	USA	Experimental/interventional study	To examine the effect of AMT on mood following a first-time ischemic stroke	Acute Stroke	Hospital
[79]	Finland	Experimental/interventional study	To determine whether everyday music listening can facilitate the recovery of cognitive functions and mood after stroke	Acute/Early Subacute Stroke including Aphasia	Hospital
[80]	UK	Analytic observational study	To assess the feasibility and acceptability of an NMT service	Early Subacute Stroke, ABI (99), Healthy (40)	Neurorehabilitation Center
[81]	Indonesia	Experimental/interventional study	To determine the effectiveness of instrumental music therapy in reducing depressive symptoms in stroke patients	Acute Stroke with Depression	Hospital
[82]	Australia	Experimental/interventional study	To explore the effects of group singing on people with aphasia	Chronic Stroke with Aphasia	Community
[83]	UK	Analytic observational study	To assess the feasibility and acceptability of delivering NMT in a neurorehabilitation center	Late Subacute Stroke (27), Other (25), Healthy (14)	Neurorehabilitation Center—Inpatients
[84]	Germany	Experimental/interventional study	To explore the potential of synchronized music playing to improve fine motor rehabilitation and mood	Early Subacute Stroke (24 IS, 4 HS; 12 RH, 12 LH)	Neurorehabilitation Center—Early rehabilitation
[85]	Italy	Experimental/interventional study	To investigate the effectiveness of a negative mismatch-based therapy on disability and QoL in patients with stroke in the subacute phase	Early and Late Subacute Stroke (9 RH, 21 LH; 21 IS, 9 HS)	Rehabilitation Center—Subacute Phase Inpatients
[86]	USA	Experimental/interventional study	To investigate the relationship between changes in mood and behavior and the number and setting of MT sessions received by people who have had astroke or TBI	Stroke and TBI with Depression	Rehabilitation Center—Inpatients
[87]	China	Experimental/interventional study	To compare the effects of MIT and speech therapy on patients with non-fluent aphasia	Early Subacute Stroke with Aphasia (24 LH, IS; 16 LH, HS)	Rehabilitation Center—Inpatients
[88]	China	Experimental/interventional study	To investigate the impact of music kinetic and exercise therapies on the depression level of elderly patients undergoing post-stroke rehabilitation	Early Subacute Stroke	Hospital—Inpatients

**Table 3:** Summary of characteristics of study participants.

Citation	*n*	Mean Age (Years)	Gender (M/F)	Education	Time Post-Stroke
[40]	47	49.6 (18.5)	(21/26)	Elementary school: (*n* = 7, 22%); high school (*n* = 12, 39%); university (*n* = 12, 39%)	391.1 (309.2) days, range (23–1208)
[41]	56	64.15 (11.65)	(37/19)	11.50 (10.00, 15.00) (median, IQR)	N/S
[42]	72	64 (11.60)	(45/27)	11, range (10–15)	<14 days
[43]	123	62.7 (6.70)	(69/54)	14.2 (4.1)	969.8 (422.9) days
[44]	20	59.8 (11.70)	(12/8)	N/S	≥6 months
[45]	84	59.9 (11.80)	(46/38)	Literacy (*n* = 7); elementary school (*n* = 51); high school/university (*n* = 25)	N/S
[46]	30	61.30 (8.29)	(22/8)	N/S	2 (1.05) months
[47]	23	62.9 (12.50)	(11/12)	N/S	5.75 (3.76) years
[48]	25	56.7, range (35–72)	(8/17)	N/S	7 days
[49]	65	75.01 (4.0)	(33/32)	N/S	N/S
[50]	28	64.2 (9.41), range (44–79)	(20/8)	15.2 (2.4), range (10–21)	6.1(6.6) years, range (1.1–21.9)
[51]	39	60.1 range (45–74)	(23/16)	N/S	65.8 days, range (32–162)
[52]	30	54.7 (10.76)	(16/14)	16.23 (2.58)	66.9 (14.41) months
[53]	38	76 (11.80)	(19/19)	N/S	<7 days
[54]	33	58 (7.12)	(23/10)	Elementary/high school (*n* = 8, 50%); university degree (n = 8, 50%)	≥6 months
[55]	15	51.9 (11.02)	(13/2)	High school (*n* = 7, 46.7%); university (*n* = 8, 53.3%)	10.25 (6.85) years
[56]	30	60.7 (8.59)	(15/15)	Elementary/high school (*n* = 14, 93.3%); university (*n* = 1, 6.7%)	<14 days
[57]	18	51.7 (13.50)	(17/1)	N/S	<6 months
[58]	44	62.4 (13.51)	(26/18)	N/S	N/S
[59]	92	72.9 (10.20)	(44/48)	N/S	<6 months
[60]	14	43.92 (10.41)	(9/5)	13.85 (2.53), range (12–21)	21.93 (10.53) years, range (7–40)
[61]	14	N/S	N/S	N/S	N/S
[62]	40	59 (12)	(30/10)	N/S	N/S
[63]	18	59.89 (16.30)	(6/12)	N/S	N/S
[64]	30	61.49 (10.94)	(16/14)	N/S	20.68 (25.19) months
[65]	30	54, range (45–69)	(17/13)	N/S	>6 months
[66]	45	63, range (48–72)	(17/28)	12.7, range (7–18)	123 days
[67]	44	56, range (25–69)	(33/11)	13.7, range (6–17)	148.4 days
[68]	1	50	(0/1)	N/S	N/S
[69]	61	64, range (44–84)	(29/32)	N/S	N/S
[70]	15	65 (6.26), range (51–74)	(8/7)	N/S	N/S
[71]	13	52 ± 14, range (21–68)	(9/4)	N/S	46.4 (36.5) months, range (8–144)
[72]	20	66.1, range (61–89)	(14/6)	10.4 (4.67), range (2–17)	3.4 (4.1) years
[73]	38	70.4 (8.9)	(16/22)	None (*n* = 6, 31.6%); elementary (*n* = 6, 31.6%); high school (*n* = 6, 31.6%); university (*n* = 1, 5.4%)	<8 weeks
[74]	65	62.4 (8.9)	(35/30)	N/S	range (12–180) days
[75]	20	59.1 (9.04)	(17/3)	9.4 (5.3)	26.22 (22.92) months, range (6.5–74)
[76]	5	40.8 (8.73), range (29–51)	(5/0)	Elementary (*n* = 1); high school (*n* = 1); university (*n* = 3)	126 (115) days, range (31–322)
[77]	20	51.02, range (24–79)	(9/11)	N/S	4.4 (4.65) years, range (1–14)
[78]	44	67.77 (12.19)	(21/23)	N/S	4.71 (3.23) days
[79]	55	57.7 (8.95)	(29/26)	10.9 (3.53)	8.73 (3.87) days
[80]	139	73.23 (16.67)	(31/77)	N/S	25.9 (14.03) days
[81]	59	50, range (30–74)	(30/29)	N/S	5 days, range (1–20)
[82]	13	58.3 (13.8)	(10/3)	High school (*n* = 6, 46%); certificate (*n* = 3, 23%); university (*n* = 4, 31%)	N/S
[83]	66	68.7 (17.5)	(25/24)	N/S	137.6 (108.8) days
[84]	28	66.35 (11.15)	(12/16)	N/S	43.25 (27.75) days
[85]	30	57.53 (13.33)	(13/17)	N/S	<6 months
[86]	10	60.5 (13.5)	(4/6)	N/S	N/S
[87]	40	53.07 (9.95)	(31/9)	N/S	2.27 (1.56) months
[88]	65	81.14 (8.33)	(28/37)	N/S	14 days

N/S (not specified).

**Table 4:** Description of music interventions for adults with stroke.

Citation	Types of Music Interventions	Intervention Modality	Intervention Description	Interventionist	Comparator	Setting of Intervention Sessions	Number of Sessions	Duration of Sessions (min)	Intervention Period (Weeks)
[40]	Music Therapy Intervention	Active	Therapeutic songwriting	Music therapist	Standard care	Group	12	60	6
[41]	Music-Based Intervention	Receptive	Music listening with mindfulness	Assistant psychologist	(A) Music listening alone (B) Audiobook listening	Group	40	60	8
[42]	Music-Based Intervention	Receptive	Music listening with mindfulness	Assistant psychologist	(A) Music listening alone (B) Audiobook listening	Group	40	60	8
[43]	Music-Based Intervention	Active	R-MT—listening to music while performing coordinated rhythmic and cognitively demanding movements	Therapists, researchers	(A) Horse-riding. (B) R-MT with 1 year of delay	Group	24	N/S	12
[44]	Music Therapy Intervention	Active	Neurologic Music Therapy—RAS	Music specialist, researchers	Gait training alone	Group	30	30	6
[45]	Music Therapy Intervention	Receptive	Listening to classical music, sharing experiences, breathing exercises	N/S	Healthy participants	Group	1	50	1
[46]	Music-Based Intervention	Receptive	Listening to Western music while exercising	N/S	Listening to non-Western music while exercising	N/S	10	60	2
[47]	Music Therapy Intervention	Active	Community choir	Music therapist	None	Group	1× week	N/S	N/S
[48]	Music Therapy Intervention	Receptive	Listening to self-selected music	Self-administered	None	Group	20	60	8
[49]	Music-Based Intervention	Receptive	Music-guided exercise	Research assistant	Standard care	Group	96	45	24
[50]	Music-Based Intervention	Active	Music-supported upper limb rehabilitation	Music therapist and occupational therapist	Conventional physical training	Group	30	N/S	10
[51]	Music-Based Intervention	Active	Music-supported upper limb rehabilitation	Occupational therapist	Standard care	Individual	20	30	8
[52]	Music Therapy Intervention	Active	Neurologic Music Therapy—TIMP	(Neurologic) music therapist	(A) Combination of TIMP with CMI. (B) Motor imagery without cues	Group	9	45	6
[53]	Music-Based Intervention	Receptive	Listening to preferred music	Staff, patient, and family	Standard care	Group	70	60	12
[54]	Music Therapy Intervention	Active	Neurologic Music Therapy—RAS	N/S	Standard care	Group	16	N/S	8
[55]	Music Therapy Intervention	Active	Neurologic Music Therapy—MACT	(Neurologic) music therapist	Nonmusical APT	Individual	3	45	3
[56]	Music Therapy Intervention	Active	Music-guided exercise	Music therapist and researcher	Standard care	Group	24	60	8
[57]	Music Therapy Intervention	Active	Playing, singing, speaking	N/S	Standard care	Group	8	40	4
[58]	Music-Based Intervention	Receptive	Listening to self-selected music	N/S	Daily activities	Individual	1	60	1
[59]	Music Therapy Intervention	Receptive	Music listening and acupuncture	Health professional	(A) Needling and acupoint injection. (B) Standard treatment	Individual	15	20	3
[60]	Music Therapy Intervention	Active	Neurologic Music Therapy—MEFT	(Neurologic) music therapist	(A) Singing group. (B) Standard care	Group	5	60	1
[61]	Music Therapy Intervention	Active	Music playing, singing, speaking	Music therapist	None	Individual	4	N/S	2
[62]	Music Therapy Intervention	Active	Music playing, singing, speaking	Music therapist	None	Individual and group	At least 1	N/S	N/S
[63]	Music Therapy Intervention	Active	Music playing, singing, speaking	Music therapist	Standard care	Group	10	N/S	4
[64]	Music Therapy Intervention	Active	Nordoff Robbins Music Therapy	Music therapist	HEP	Group	12	45	6
[65]	Music-Based Intervention	Active	AMST + CMDT	N/S	CMDT only	Group	18	30	6
[66]	Music Therapy Intervention	Active	Music playing, music listening	Music therapist	None	Individual	2	90	4
[67]	Music Therapy Intervention	Active	Music playing, music listening	Music therapist	None	Individual	1	90	1
[68]	Music Therapy Intervention	Active	Music playing, singing, speaking	Music therapist	None	Individual	12	N/S	3
[69]	Music Therapy Intervention	Receptive	GIM, cognitive music Therapy	Music therapist	Standard care	Individual	10	N/S	5
[70]	Music-Based Intervention	Active	R-MT	Instructor of R-MT	None	Group	24	60	12
[71]	Music Therapy Intervention	Active	Playing musical instruments for upper limb rehabilitation	Music therapist	None	Group	12	45	6
[72]	Music Therapy Intervention	Active	Musical improvisation, singing, vocalizing	Music therapist	Speech therapy only	Individual	30	30	15
[73]	Music Therapy Intervention	Active	RAMT	Music therapist	Standard care	Group	20	30	7
[74]	Music-Based Intervention	Active	Upper extremity treatment with sonification techniques	Physiotherapist or occupational therapist	Standard upper extremity rehabilitation	Group	20	35	4
[75]	Music-Based Intervention	Active	MST	Neuropsychologist with musical training	Healthy control group	Group	20	30	4
[76]	Music Therapy Intervention	Active	Therapeutic songwriting	Music therapist	None	Group	12	N/S	6
[77]	Music Therapy Intervention	Active	FMT—Execution of musical instruments	Music therapist	None	Individual	20	20	20
[78]	Music Therapy Intervention	Active	Mixed neuroinformatic approach—singing, improvising, activating consonant music	Music therapist	None	Individual	At least 1	25	N/S
[79]	Music Therapy Intervention	Receptive	Listening to preferred music	Music therapist	(A) Listening to audiobook (B) No listening material	Group	40	60	8
[80]	Music Therapy Intervention	Active	Neurologic Music Therapy	Music therapist	None	Individual or group	Average of 4.8 sessions	N/S	Max. 24 months
[81]	Music Therapy Intervention	Receptive	Listening to instrumental music	Primary caregiver	(A) Standard care without music. (B) Combined treatment	N/S	N/S	30	N/S
[82]	Music Therapy Intervention	Active	Community choir	Music therapist	None	Group	24	120	6 months
[83]	Music Therapy Intervention	Active	Neurologic Music Therapy	Music therapist	None	Individual	At least 1	N/S	15 months
[84]	Music-Based Intervention	Active	Music-supported motor training—synchronic instrumental playing in pairs	N/S	In-turn instrumental playing in pairs	Group	10	30	4
[85]	Music-Based Intervention	Receptive	Identifying mismatch in music	N/S	Standard care	Group	12	20	4
[86]	Music Therapy Intervention	Active	Improvising, singing, composing, playing	Music therapist	None	Individual and group	4 to 10	30 to 40	1 to 3
[87]	Music Therapy Intervention	Active	Neurologic Music Therapy—MIT	Music therapist	Speech therapy only	Individual	20	30	8
[88]	Music-Based Intervention	Receptive	Music-supported therapy (physical exercises with background music)	Physiotherapist	Physical exercise only	Group	80	30	8

N/S (not specified).

**Table 5:** Study outcomes.

Citation	Main Outcomes		Other Primary Outcomes						Main Findings	Results
	Executive Functions	Emotional Well-Being	Cognitive Domain	Emotional Domain	Quality of Life	Motor Domain	Functional Domain	Musical Functions		
[40]		* Self-concept and well-being (HISDS; SWLS, PHQ-9; ERQ)							Improvements in satisfaction of life; no significant results for self-concept and emotional regulation measures	Mixed
[41]		* Participants’ experiences with mood, emotional needs, and social interaction (Kruskal–Wallis test)							Improvements in mood, level of activity, and cognition	Positive
[42]	Verbal memory, speed of processing (BMIPB); working memory (DST; SPT)	* Mood (HADS)	* Cognition (MOCA); attention (TEA)						Improvements in mood, level of activity, and cognition	Positive
[43]		* Perceived recovery (SIS)	BNIS			TUG; BBS; BDL-BS; Grippit			The perception of stroke recovery was higher among R-MT and horse-riding therapy participants compared to controls Improvements were sustained until 6 months later, and corresponding gains were observed for the secondary outcomes	Positive
[44]					SS-QOL	* Gait (BBS)			Improvements in balance, gait performance, and QoL	Positive
[45]				* Anxiety (STAI)			FIM		Improvement in anxiety levels in both groups; no significant difference between the groups	Positive
[46]			* Cognitive state (MMSE)	* Depression (BDI), sleep quality (PSQI), anxiety (BAI)					There was no statistical difference between the three groups in pre- or post-treatment results	Negative
[47]		* Mood, social interaction, self-management (WHOQOL-BREF; SIPSO)							Positive experiences in self-management, social interaction, mood, and communication	Positive
[48]		* Mood, emotional needs, social interaction (interview)							Positive experiences in relaxation, mood, physical and cognitive recovery	Positive
[49]			* Cognitive recovery (MMSE)			BI, CBF			Improvements in mood profile of stroke patients, and higher recovery rate	Positive
[50]	* Cognitive flexibility; verbal fluency, working memory (TMT; D-KEFS)	* Mood (PANAS)			* SIS	* CMSA; ARAT; BBT		MET	Improvements in motor functions, in SIS for emotion, communication, and in measures for executive functions. Results confirmed previous findings and expanded the potential usage of MST for enhancing QoL	Positive
[51]	Working memory, attention, response inhibition, processing speed, mental flexibility (DST; ST; TMT)	Mood (POMS; BDI; PANAS; AES)	Memory (RAVLT)		SS-QOL; SF36	* Upper limbs (ARAT)		MRQ	Both groups improved in the motor domain, but only the music group improved in QoL. Intrinsic motivation in music was associated with better motor improvement	Positive
[52]	Mental flexibility, short-term memory (TMT-B; DST)	Self-efficacy (GSE), affective state (MAACL; SAM)				* Upper limbs (FM-UE; WMFT; MAL)			Significant improvements in motor domain. TIMP + MI positive for mental flexibility; active TIMP interventions enhance positive affect	Positive
[53]			* Attention, memory, language (COGNISTAT),	* Depression, anxiety (HADS)	SAQOL-39	Disability (MRS)	FIM		Intervention was experienced as positive but no improvements in outcome measures were shown	Negative
[54]		Mood (POMS), relationships (RCS)			SS-QOL	* ROM; BST			Improvements in range of motion, flexibility, and mood; increased frequency and quality of interpersonal relationships	Positive
[55]	* Attention control TMT A + B; BPT		divided attention (DS)	Likert scales of effort and motivation					Greater improvements on TMT-B for the experimental group than for the control group	Positive
[56]		Mood (POMS)		Depression (CES-D)		* ROM; MRC-SS	ADL (K-MBI)		Improvements in mood state and motor outcomes	Positive
[57]		* Mood (BAI; BDI)							Improvements in both outcome measures were greater in the experimental group, but only BDI scores were statistically significant	Positive
[58]				* Anxiety (STAI; HADS)					The music group showed greater improvements in scores than the control group	Positive
[59]				* Depression (HAMD)			Safety (TESS)		Scores of both measures improved in all groups but were significantly better in the MT group	Positive
[60]	* Task shifting TMT A + B; PASAT							AMMA	Pre- and post-test group differences revealed a trend toward improvement in the MT group over the singing group	Positive
[61]		* Mood (POMS-BI)							Significant differences between pre- and post-MT intervention in a positive direction were shown	Positive
[62]		* Mood, satisfaction (semi-structured interviews; VAS)					* Pain (semi-structured interviews; VAS)		Significant improvements in mood, level of satisfaction, and decrease in pain from pre- to post-MT intervention	Positive
[63]		Mood, social interaction (FS; VAMS; SIP)							Family members assessed the social interaction as higher in the MT group; staff rated participants in the MT group as more active and cooperative. Self-ratings and family ratings of mood showed improvement in the MT group. The more impaired a participant’s social behavior at the outset, the more likely the benefit from MT	Positive
[64]		Emotional and social well-being (PHQ-9; WHO-5)			QLI—stroke; SSEQ; SIS; semi-structured interviews	* FMS	Sensory impairment (SWM; MRS)		MULT-I participants showed reduced depression and improved QoL. Brain-derived neurotrophic factor levels significantly increased for MULT-I. The implementation of a music-enriched environment is feasible and reduces post-stroke depression	Positive
[65]	* Cognitive control and flexibility TMT A + B; ST; DST		* Attention, memory (SNSB)						Both groups improved in outcomes. Performance speed on the TMT-A and DST was faster in the CMDT + AMST group than in the CMDT group	Positive
[66]			* Cognitive state: attention, memory (MMSE; CLQT)					SCM	Improvements in musical activities measuring sustained and selective attention, echoic rhythmic, working memory, musical memory, and auditory learning skills	Positive
[67]	FAB, ACE-R	Behavioral observation	* Cognitive state: attention, memory (MMSE, ACE-R, CLQT)					ECMUS	Positive results on internal consistency, excellent test–retest and inter-rater reliability, and weak to strong correlations to related, non-musical constructs	Positive
[68]		* Mood (CECS; AIS; POMS); coping strategies (CISS); self-efficacy (GSE); well-being (MPQ)		* Anxiety (STAI)					Positive results in CECS, AIS, and STAI. Other outcomes remained unchanged	Mixed
[69]					* Vitality, health perception, social functioning, emotional and mental health, limitations (SF-36; SA-SIP30; The Cantril Ladder)				Improvement in QoL measures. MT did not influence the results related to pain, limitation of social roles, relationships, self-care, or mobility	Mixed
[70]		Mood, social interactions (semi-structured interviews)	Overall cognitive functioning (semi-structured interviews)						Positive perception of participants on cognitive and motor challenge, social integration, and mood. Negative experiences were associated with not being able to perform the exercises, and with group members who dominated the conversational space	Mixed
[71]		Well-being (WHO-5)			SIS	* FMS; MRS			Significant improvement in motor domain and well-being that persisted at 1 year. ADL and social participation improved only from post-intervention to 1-year follow-up. Subjects reported feelings of ownership of their impaired limb, more spontaneous movement, and enhanced emotional engagement	Positive
[72]			* Aphasia (Milan Protocol, AAT)	Psychological aspects (BDI; BFQ)	SF36				In total, 50% of participants improved in vitality scores of the SF36, but not significantly in psychological measures. The experimental group improved in spontaneous speech	Mixed
[73]				Anxiety, depression (HADS)	MQOL- It	* GPDT; 9 HPT; TUG	It-NIHSS; FIM	MBEA; MTRS	The experimental group showed greater improvement in measures of QoL, as well as a decrease in anxiety and depression. Functional and disability levels improved in both groups and motor improvements were greater in the experimental group	Positive
[74]					MQOL- It	* FMS, BBT; AS			Improvements in motor domain, but not in QoL	Mixed
[75]	Attention, working memory (DST; RAVLT; ST; TMT)	Affective state (PANAS; AES)	Global cognitive functioning (MMSE; STT; AT)	Depression (BDI)	SF36; SS-QOL	* ARAT, APS, BBT, 9 HPT, BI			The experimental group showed significant improvements in the motor domain, attention, speed of processing, rate of learning, valence of the experience, and mood	Positive
[76]		* Identity, self-concept (HISD; TSCS-2); well-being (SWLS; FS); subjective distress (GAD-7; PHQ-9; PANAS)					FIM		Greatest improvement was across self-concept and subjective well-being	Positive
[77]		Subjective emotional state (questionnaire)			Observation and interpretation	* Functional capacity (observation and interpretation)			Both groups improved in motor domain, collaboration skills, logical thinking and perception, which were partly maintained at follow-up. Both groups reported improved social life, concentration, and self-esteem	Positive
[78]		* Mood (FS)	Cognitive functioning (Mini MOCA)	Depression (PHQ-9)			NIHSS		Significant improvements in mood, depression, and cognition after AMT	Mixed
[79]	* Verbal memory (RBMT); WMS-R	* Mood (POMS)	* Attention (DST); visuospatial (CT, BVRT); aphasia (BDAE)		SAQOL-39			MBEA	The music group improved in focused attention and verbal memory after 3 months. At the 6-month stage, verbal memory recovery, and focused attention scored higher in the music group. Depression and confusion scores were significantly lower in the music group	Positive
[80]		Mood, social interaction (questionnaires, VAMS)	Communication (questionnaires)			Questionnaires			NMT services were feasible and helpful, particularly for mood, possibly improving engagement in rehabilitation	Positive
[81]				* Depression (HAMD-17)					Combined treatment provided the most significant influence on reducing depression	Mixed
[82]		* Mood (GHQ-12; VAMS); * social engagement (SOBI)	Cognitive functioning (SIS-3)						The GHQ-12 showed a reduction in psychological distress; the interviews revealed increased confidence, peer support, enhanced mood, increased motivation, and changes to communication	Positive
[83]		Mood (VAMS)				Feasibility, acceptability (questionnaires)			A one-day-per-week NMT was feasible, acceptable, and helpful, supporting patient engagement in rehabilitation exercises, mood, and motivation	Positive
[84]		Mood (POMS; FS)				* Motor (9 HPT; FTT)			Both groups showed improvements in fine motor control and reductions in depression, anxiety, and fatigue. Music-supported rehabilitation showed improvements in individuals and in patient pairs	Positive
[85]					* SS-QOL		* Disability (DRS; MBI)		Music group showed greater improvement in all outcomes compared to the control group	Positive
[86]		* Mood (FS); * social interaction (adapted SIP)							MT had a greater effect on behavioral measures than on mood. Group sessions appeared to affect social interaction and individual sessions, marginally affecting motivation for treatment	Mixed
[87]			* Aphasia (BDAE)	Anxiety (HAMA); depression (HAMD)					The MIT group improved significantly in language outcomes and in the HAMD; no significant effect on the HAMA	Mixed
[88]				* Depression (HDRS-24)			Health-specific focus (HRFS)		Both interventions decreased the level of depression, but the intervention group had a slightly better effect	Mixed

* Main study outcomes.

## Supplementary Materials

The following supporting information can be downloaded at https://www.mdpi.com/article/10.3390/neurosci5040041/s1.
