# Supplementary material for: Exploring Music-Based Interventions for Executive Functioning and Emotional Well-Being in Stroke Rehabilitation: A Scoping Review
Source: NeuroSci. 2024 Nov 27;5(4):565–99. doi: 10.3390/neurosci5040041 (PMC11677499; doi:10.3390/neurosci5040041)
Supplement: Supplementary file 1 [file neurosci-05-00041-s001.zip › neurosci-3290135-supplementary.pdf]

Supplementary Material

Literature search strategy

A summary of the search results from databases:

PubMed (NLM) from inception to 25 October 2022 (4375 Results)

CINAHL (EbscoHost) from inception to 25 October 2022 (1615 Results)

PsycInfo (EbscoHost) from inception to 25 October 2022 (938 Results)

PubMed (NLM)

("cognition disorders"[MeSH] OR "Cognition"[Mesh] OR "cognitive impair*"[tiab] OR "decision competen*"[tiab] OR "decision capac*"[tiab] OR "Cognitive Dysfunction"[Mesh] OR "neurocognitive disord*"[tiab] OR "Neurocognitive Disorders"[Mesh] OR "Aphasia"[Mesh] OR aphas*[tiab] OR "cognitive disabil*"[tiab] OR "executive funct*"[tiab] OR affect*[tiab] OR emotion*[tiab] OR apath*[tiab] OR anxiet*[tiab] OR depress*[tiab] OR "social interact*"[tiab] OR "quality of life"[tiab] OR "cognitive flexibil*"[tiab] OR "cognitive rehab*"[tiab] OR psychology[sh] OR "Prefrontal Cortex"[MeSH] OR "prefrontal cortex*"[tiab] OR "Attention"[MeSH] OR attent*[tiab] OR "Affect"[MeSH] OR "Emotions"[MeSH] OR "Executive Function"[MeSH] OR "Neurobehavioral Manifestations"[Mesh] OR "neurobehavioral manifest*"[tiab] OR "cognitive symptom*"[tiab] OR "cognitive manifest*"[tiab] OR “anxiety”[MeSH]) AND ("stroke"[MeSH Terms] OR strok*[tw] OR "Stroke rehabilitation"[MESH] OR "cerebrovascular disorders"[MESH] OR "cerebrovascular disord*"[tiab] OR "stroke rehab*"[tiab] OR "chronic strok*"[tiab] OR "cerebrovascular accid*"[tiab] OR "subarachnoid haemorrhag*"[tiab] OR “subarachnoid hemorrhag*”[tiab] OR cva[tiab] OR “cerebral infarction”[tiab] OR “brain infarction”[tiab] OR “brain ischemia”[tiab] OR “intracranial hemorrhag*”[tiab] OR “intracranial haemorrhag*”[tiab] OR “brain hemorrhag*”[tiab] OR “brain haemorrhag*” OR “intracranial embolism”[tiab] OR “brain embolism”[tiab] OR “intracranial thrombosis”[tiab] OR “brain thrombosis”[tiab] OR “cerebral embolism”[tiab] OR “cerebral thrombosis”[tiab] OR “brain ischemia”[mesh] OR “intracranial hemorrhages”[mesh] OR “intracranial embolism and thrombosis”[mesh]) AND ("Music Therapy"[MeSH] OR "Acoustic Stimulation"[MeSH] OR sing*[tiab] OR "auditory stimul*"[tiab] OR "music therap*"[tiab] OR improvis*[tiab] OR songwrit*[tiab] OR song*[tiab] OR "patterned sensory enhanc*"[tiab] OR "melodic intonation therap*"[tiab] OR "rhythmic speech*"[tiab] OR "vocal intonation therap*"[tiab] OR (music*[tiab] AND (therap*[tiab] OR "based-intervent*"[tiab] OR listen*[tiab] OR prefer*[tiab] OR relax*[tiab] OR play*[tiab] OR experien*[tiab] OR therapeutic*[tiab] OR instrument*[tiab] OR perform*[tiab] OR "neglect train*"[tiab] OR "attentional control train*"[tiab] OR expos*[tiab] OR orient*[tiab] OR neurolog*[tiab] OR recept*[tiab] OR intervent*[tiab])) AND (double-blind method[mh] OR single-blind method[mh] OR clinical trial[pt] OR "clinical trial"[tw] OR "pragmatic trial"[tw]

OR "real world trial"[tw] OR ((singl*[tw] OR doubl*[tw] OR trebl*[tw] OR tripl*[tw]) AND (mask*[tw] OR blind*[tw])) OR "latin square"[tw] OR placebos[mh] OR placebo*[tw] OR random*[tw] OR research design[mh:noexp] OR comparative study[pt] OR evaluation studies[pt] OR follow-up studies[mh] OR prospective studies[mh] OR cross-over studies[mh] OR control*[tw] OR prospectiv*[tw] OR volunteer*[tw] OR "Clinical Trial, Phase III"[Publication Type] OR "Clinical Trial, Phase II"[Publication Type] OR "Clinical Trial, Phase I"[Publication Type] OR "Controlled Clinical Trial"[Publication Type] OR "Feasibility Studies"[Mesh] OR "feasibility stud*"[tiab] OR "Pilot Projects"[Mesh] OR "pilot project*"[tiab] OR "pilot stud*"[tiab]) NOT ((infant[mh] OR child[mh] OR adolescent[mh]) NOT (adult[mh])) NOT (animals[MeSH] NOT humans[MeSH]))

APA PsycInfo (EbscoHost)

S1	(TI ("cognitive impair*" OR "decision competen*" OR "decision capac*" OR "neurocognitive disord*" OR aphas* OR "cognitive disabil*" OR "executive funct*" OR affect* OR emotion* OR apath* OR anxiet* OR depress* OR "social interact*" OR "quality of life" OR "cognitive flexibil*" OR "cognitive rehab*" OR "prefrontal cortex*" OR attent* OR "Neurobehavioral Manifest*" OR "cognitive symptom*" OR "cognitive manifest*")) OR (AB ("cognitive impair*" OR "decision competen*" OR "decision capac*" OR "neurocognitive disord*" OR aphas* OR "cognitive disabil*" OR "executive funct*" OR affect* OR emotion* OR apath* OR anxiet* OR depress* OR "social interact*" OR "quality of life" OR "cognitive flexibil*" OR "cognitive rehab*" OR "prefrontal cortex*" OR attent* OR "Neurobehavioral Manifest*" OR "cognitive symptom*" OR "cognitive manifest*")) OR (DE ("cognitive impairment" OR DE "Cognition" OR DE "Neurocognitive Disorders" OR DE "Aphasia" OR DE psychology OR DE "Prefrontal Cortex" OR DE "Attention" OR DE "emotional regulation" OR DE "Emotions" OR DE "Executive Function" OR DE "anxiety")))
S2	(TI (strok* OR "cerebrovascular disord*" OR "stroke rehab*" OR "chronic strok*" OR "cerebrovascular accid*" OR "subarachnoid haemorrhag*" OR "subarachnoid hemorrhag*" OR cva OR "cerebral infarction" OR "brain infarction" OR "brain ischemia" OR "intracranial hemorrhag*" OR "intracranial haemorrhag*" OR "brain hemorrhag*" OR "brain haemorrhag*" OR "intracranial embolism" OR "brain embolism" OR "intracranial thrombosis" OR "brain thrombosis" OR "cerebral embolism" OR "cerebral thrombosis") OR (AB (strok* OR "cerebrovascular disord*" OR "stroke rehab*" OR "chronic strok*" OR "cerebrovascular accid*" OR "subarachnoid haemorrhag*" OR "subarachnoid hemorrhag*" OR cva OR "cerebral infarction" OR "brain infarction" OR "brain ischemia" OR "intracranial hemorrhag*" OR "intracranial haemorrhag*" OR "brain hemorrhag*" OR "brain haemorrhag*" OR "intracranial embolism" OR "brain embolism" OR "intracranial thrombosis" OR "brain thrombosis" OR "cerebral embolism" OR

	“cerebral thrombosis”) OR (DE (“cerebrovascular accidents” OR "cerebrovascular disorders" OR "brain injuries" OR "Cerebral Ischemia")))
S3	(TI (sing* OR "Auditory stimul*" OR "music therap*" OR improvis* OR songwrit* OR song* OR "patterned sensory enhanc*" OR "melodic intonation therap*" OR "rhythmic speech*" OR "vocal intonation therap*" OR ((music*) AND (therap* OR "based-intervent*" OR listen* OR prefer* OR relax* OR play* OR experient* OR therapeutic* OR instrument* OR perform* OR "neglect train*" OR "attentional control train*" OR expos* OR orient* OR neurolog* OR recept* OR intervent*)) OR (AB (sing* OR "Auditory stimul*" OR "music therap*" OR improvis* OR songwrit* OR song* OR "patterned sensory enhanc*" OR "melodic intonation therap*" OR "rhythmic speech*" OR "vocal intonation therap*" OR ((music*) AND (therap* OR "based-intervent*" OR listen* OR prefer* OR relax* OR play* OR experient* OR therapeutic* OR instrument* OR perform* OR "neglect train*" OR "attentional control train*" OR expos* OR orient* OR neurolog* OR recept* OR intervent*)) OR (DE (“music” OR DE "Music Therapy" OR DE "Auditory Stimulation"))))
S4	(TI (“clinical trial*" OR "pragmatic trial*" OR "real world trial*" OR ((singl* OR doubl* OR trebl* OR tripl*) AND (mask* OR blind*)) OR “latin square*” OR placebo* OR random* OR “comparative stud*” OR “evaluation stud” OR control* OR prospectiv* OR volunteer* OR "Clinical Trial Phase III" OR "Clinical Trial Phase II" OR "Clinical Trial Phase I" OR “controlled trial*” OR “control trial*” OR "feasibility stud*" OR "pilot project*" OR “pilot stud*”) OR (AB (“clinical trial*" OR "pragmatic trial*" OR "real world trial*" OR ((singl* OR doubl* OR trebl* OR tripl*) AND (mask* OR blind*)) OR “latin square*” OR placebo* OR random* OR “comparative stud*” OR “evaluation stud” OR control* OR prospectiv* OR volunteer* OR "Clinical Trial Phase III" OR "Clinical Trial Phase II" OR "Clinical Trial Phase I" OR “controlled trial*” OR “control trial*” OR "feasibility stud*" OR "pilot project*" OR “pilot stud*”) OR (DE ("Experiment Controls" OR “Clinical Trials” OR “Randomized Controlled Trials” OR placebo OR “experimental design” OR followup studies” OR “prospective studies” OR “longitudinal studies”))))
S5	S1 AND S2 AND S3 AND S4
S6	S5 NOT (((DE "Animal Research") OR (DE "Vivisection") OR (TI "animal model*") OR (TI “animal”)) NOT ((TI "human*") OR (AB “human*”)))

CINAHL (EbscoHost)

S1	(TI ("cognitive impair*" OR "decision competen*" OR "decision capac*" OR "neurocognitive disord*" OR aphas* OR "cognitive disabil*" OR "executive funct*" OR affect* OR emotion* OR apath* OR anxiet* OR depress* OR "social interact*" OR "quality of life" OR "cognitive flexibil*" OR "cognitive rehab*" OR "prefrontal cortex*" OR attent* OR "Neurobehavioral Manifest*" OR "cognitive symptom*" OR "cognitive manifest*")) OR (AB ("cognitive impair*" OR "decision competen*" OR "decision capac*" OR "neurocognitive disord*" OR aphas* OR "cognitive disabil*" OR "executive funct*" OR affect* OR emotion* OR apath* OR anxiet* OR depress* OR "social interact*" OR "quality of life" OR "cognitive flexibil*" OR "cognitive rehab*" OR "prefrontal cortex*" OR attent* OR "Neurobehavioral Manifest*" OR "cognitive symptom*" OR "cognitive manifest*")) OR (MH ("mild cognitive impairment" OR "Cognition" OR "cognition Disorders" OR "Aphasia" OR psychology OR "Prefrontal Cortex" OR "Attention" OR “emotional regulation” OR "Emotions" OR "Executive Function" OR “anxiety”)))
S2	(TI (strok* OR "cerebrovascular disord*" OR "stroke rehab*" OR "chronic strok*" OR "cerebrovascular accid*" OR "subarachnoid haemorrhag*" OR “subarachnoid hemorrhag*” OR cva OR “cerebral infarction” OR “brain infarction” OR “brain ischemia” OR “intracranial hemorrhag*” OR “intracranial haemorrhag*” OR “brain hemorrhag*” OR “brain haemorrhag*” OR “intracranial embolism” OR “brain embolism” OR “intracranial thrombosis” OR “brain thrombosis” OR “cerebral embolism” OR “cerebral thrombosis”)) OR (AB (strok* OR "cerebrovascular disord*" OR "stroke rehab*" OR "chronic strok*" OR "cerebrovascular accid*" OR "subarachnoid haemorrhag*" OR “subarachnoid hemorrhag*” OR cva OR “cerebral infarction” OR “brain infarction” OR “brain ischemia” OR “intracranial hemorrhag*” OR “intracranial haemorrhag*” OR “brain hemorrhag*” OR “brain haemorrhag*” OR “intracranial embolism” OR “brain embolism” OR “intracranial thrombosis” OR “brain thrombosis” OR “cerebral embolism” OR “cerebral thrombosis”) OR (MH (“stroke” OR "cerebrovascular disorders" OR "brain injuries" OR "Cerebral Ischemia+" OR "Intracranial Hemorrhage+" OR "Intracranial Embolism and Thrombosis+")))
S3	(TI (sing* OR "auditory stimul*" OR "music therap*" OR improvis* OR songwrit* OR song* OR "patterned sensory enhanc*" OR "melodic intonation therap*" OR "rhythmic speech*" OR "vocal intonation therap*" OR ((music*) AND (therap* OR "based-intervent*" OR listen* OR prefer* OR relax* OR play* OR experien* OR therapeutic* OR instrument* OR perform* OR "neglect train*" OR "attentional control train*" OR expos* OR orient* OR neurolog* OR

	recept* OR intervent*)) OR (AB (music* OR sing* OR "Auditory stimul*" OR "music therap*" OR improvis* OR songwrit* OR song* OR "patterned sensory enhanc*" OR "melodic intonation therap*" OR "rhythmic speech*" OR "vocal intonation therap*" OR ((music*) AND (therap* OR "based-intervent*" OR listen* OR prefer* OR relax* OR play* OR experient* OR therapeutic* OR instrument* OR perform* OR "neglect train*" OR "attentional control train*" OR expos* OR orient* OR neurolog* OR recept* OR intervent*)) OR (MH (“music” OR "music therapy" OR "acoustic stimulation”))))
S4	(TI (“clinical trial*" OR "pragmatic trial*" OR "real world trial*" OR ((singl* OR doubl* OR trebl* OR tripl*) AND (mask* OR blind*)) OR “latin square*” OR placebo* OR random* OR “comparative stud*” OR “evaluation stud” OR control* OR prospectiv* OR volunteer* OR "Clinical Trial Phase III" OR "Clinical Trial Phase II" OR "Clinical Trial Phase I" OR “controlled trial*” OR “control trial*” OR "feasibility stud*" OR "pilot project*" OR “pilot stud*”) OR (AB (“clinical trial*" OR "pragmatic trial*" OR "real world trial*" OR ((singl* OR doubl* OR trebl* OR tripl*) AND (mask* OR blind*)) OR “latin square*” OR placebo* OR random* OR “comparative stud*” OR “evaluation stud” OR control* OR prospectiv* OR volunteer* OR "Clinical Trial Phase III" OR "Clinical Trial Phase II" OR "Clinical Trial Phase I" OR “controlled trial*” OR “control trial*” OR "feasibility stud*" OR "pilot project*" OR “pilot stud*”) OR (MH ("double-blind studies" OR “single-blind studies” OR “Clinical Trials” OR “Randomized Controlled Trials” OR placebos OR “study design” OR “prospective studies”))))
S5	S1 AND S2 AND S3 AND S4
S6	S5 NOT (((MH "Animals+") OR (MH "Animal Studies") OR (TI "animal model*")) NOT (MH "human"))

Clinicaltrials.gov

music | stroke OR "cerebrovascular disorder" | Completed Studies<br>Limit: Completed studies

Dissertations and Theses (ProQuest)

S1	ti ("cognitive disabil*" OR "cognitive impair*" OR "executive funct*") OR ab("cognitive disabil*" OR "cognitive impair*" OR "executive funct*") OR (MAINSUBJECT “cognitive function”)
S2	ti (strok* OR "cerebrovascular disord*") OR ab(strok* OR "cerebrovascular disord*") OR (MAINSUBJECT stroke)
S3	ti(music*) OR ab(music*) OR (MAINSUBJECT music)
S4	S1 AND S2 AND S3

TRIP medical database (tripdatabase.com)

(“cognitive disabil*” OR “cognitive impair*” OR “executive funct*”) AND (strok* OR "cerebrovascular disord*”) AND (music*)

Table 2 shows the general description of the selected studies. The 49 papers included in this scoping review were published between 2000 and 2022, while the majority were published between 2016 and 2021 (61.22%, *n* = 30) (see Table 2). The first authors originated mainly from the United States (14.28%, *n* = 7), followed by the United Kingdom, Korea, and Italy (8.16%, *n* = 4 per country) (Table 3). Thirty-two were music therapy studies (65.3%) with the remainder being MBI studies (see Table 4). Study designs included 1 case report, 5 descriptive observational studies, 6 analytic observational studies, and 37 experimental/interventional studies, out of which 54% (*n* = 20) were randomized controlled trials (RCTs).

Description of music interventions for adults with stroke.

Summary of characteristics of study participants.

Participants included people diagnosed with stroke (*n* = 1663), among a population with diverse neurological diseases (*n* = 1753). Healthy participants (*n* = 201) were included in studies as control groups or were interviewed about the patient’s experiences with the received stroke treatment. The mean sample size of studies included was 33.73 (range 1–166), and the majority (40.81%, *n* = 20) involved between

20 and 40 participants. Further, 34.69% (n = 17) of studies involved less than 20 participants, and 26.53% (n = 13) involved more than 40 participants. Demographics reporting varied greatly throughout the publications, where age and gender were the characteristics most consistently informed. The average age of participants was 60.42 years (SD = 11.14), 55.78% (n = 1089) were male, and level and years of education were recorded across 38.78% (n = 19) of publications, which impeded the authors in calculating an average of years of education. Time since stroke was reported in 75.51% (n = 37), ranging from 5 days to 10.5 years, and again, the different ways of reporting made it impossible to calculate an average time. Among those, 32.43% (n = 12) included participants in the chronic stage of recovery, 29.73 % (n = 11) in the late subacute stage, 24.32% (n = 9) in the early subacute stage, and 13.51% (n = 5) in acute recovery. The type of stroke (ischemic or hemorrhagic) and the localization (left or right brain hemisphere, or multi-territorial) were reported only in 42.86% (n = 21) of the studies, including a total of 719 participants. Among those, 53.30% of the participants had left-hemisphere strokes, and 76.49% were of ischemic etiology. The cultural background of participants was only reported in 20.40% (n = 10) of studies (see Table 3).

Research Design

As displayed in Table 4, the type of research designs described varied significantly. The pre/post-tests, which compare the person's status prior to and after the intervention, accounted for 32.65% (n = 16). Two studies used healthy controls to compare the effect of the musical intervention. Furthermore, a number of studies compared the effect of the music intervention to other interventions. There were two main groups of comparators, one being "standard care" (n = 12, 29.49%) and the other being non-musical interventions (n = 12). Only 14.28% (n = 7) that used other music interventions as comparators, including listening to other types of music, a variation in the investigated music intervention, or an auditory comparator, such as audiobook listening.

Interventionists, Settings, and Units of delivery

Overall, more than half of the interventionists were music therapists (n = 26, 53.06%). Interventionists were not reported in nine studies (18.37%), and eight studies

identified them as healthcare professionals from different specialties (16.32%). Other studies included researchers (*n* = 2), caregivers (*n* = 2), a trained instructor (*n* = 1), or the participants themselves (*n* = 1) as interventionists. Out of the 49 studies, only 3 included a multidisciplinary team composed of music therapists and occupational therapists. Most studies were conducted in rehabilitation centers (57.14%, *n* = 28), including specialized neurorehabilitation centers, acute stroke units, and one residential rehabilitation facility. Other studies were conducted in general hospitals or neurology departments of hospitals (26.53%, *n* = 13), or with community-dwelling participants (16.32%, *n* = 8) (see Table 4). Interventions were offered primarily as group sessions (*n* = 30, 61.22%), while individual sessions were less frequent (*n* = 14, 28.57%). In two studies, interventions were offered in a combination of individual and group settings, while another described them as group or individual settings. Lastly, two studies did not document the treatment setting.
